# Supplementary material for: Experimental evolution supports the potential of neonicotinoid-pyrethroid combination for managing insecticide resistance in malaria vectors
Source: Sci Rep. 2021 Sep 30;11:19501. doi: 10.1038/s41598-021-99061-x (PMC8484614; doi:10.1038/s41598-021-99061-x)
Supplement: Supplementary file 1 — Supplementary Information 1. [file 41598_2021_99061_MOESM1_ESM.pdf]

Supplementary Fig. S1. Resistance of the parental Tiassalé-S line to insecticides commonly used for vector control. Insecticide susceptibility tests were performed using WHO test tubes equipped with papers impregnated with 0.05% deltamethrin, 0.5% bendiocarb, 1% fenitrothion and 4% DDT. Mortality rates are expressed as mean mortality ± 95% Wald confidence interval.

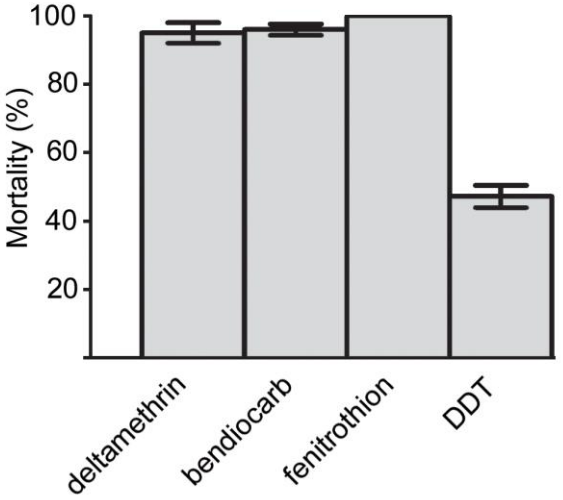

Supplementary Table S2. Ace1 genotyping data

Lines	Susceptible (119GG)	Heterozygote (119GS)	Resistant (119SS)	N	f(GG)	f(GS)	f(SS)	Chi2 P value[1]	f(S)	Chi2 P value [1]
Tiassale-S G0	22	1	2	25	78,6	3,6	7,1	-	0,10	-
Delta-R G17	27	2	1	30	90,0	6,7	3,3	0,695 (ns)	0,07	0.73 (ns)
Clothia-R G17	21	8	0	29	70,0	27,6	0,0	0,028 (*)	0,14	0.565 (ns)
Fludo-R G17	25	4	1	30	86,2	13,8	3,4	0,389 (ns)	0,10	1 (ns)

[1] Chi^2 tests between each selected line at G17 and the Tiassale-S line at G0 were performed on raw genotype and frequency data. ns: P > 0.05, * P<0.05.

Supplementary Table S3. Transcription data obtained for all genes detected by RNA-seq. See separated supplementary file.

Supplementary Fig. S4. **Overview of genes differentially transcribed in the three selected lines.** The number of genes is indicated for each line. Numbers within brackets refer to candidate genes potentially involved in insecticide resistance (see methods).

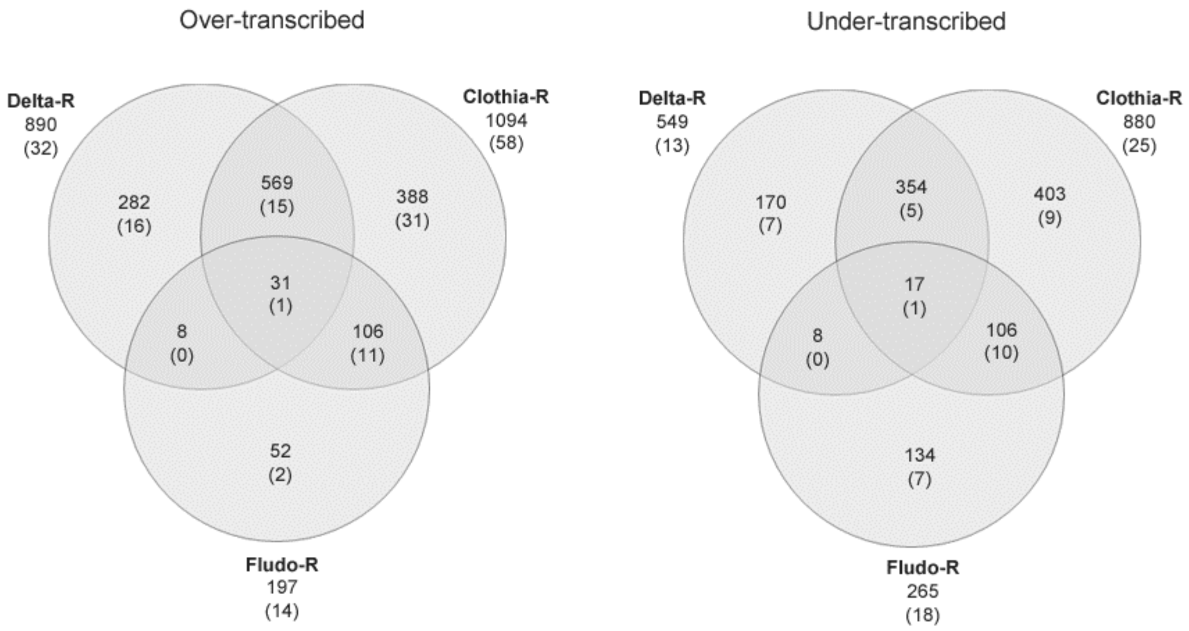

Supplementary Fig. S5. Overview of GO term enrichment analysis. Functional pathways enrichment analyses were based on genes significantly over- and under-transcribed in each selected line as compared to Tiassalé-S line using DAVID functional annotation tool (modified Fisher's exact test with P < 0.05). Only GO terms from the « biological process » family showing an enrichment associated with a P value <0.05 and a minimum number of 9 genes are shown. Fold-enrichment (x axis), P value (color scale) and class size (dote size) are indicated.

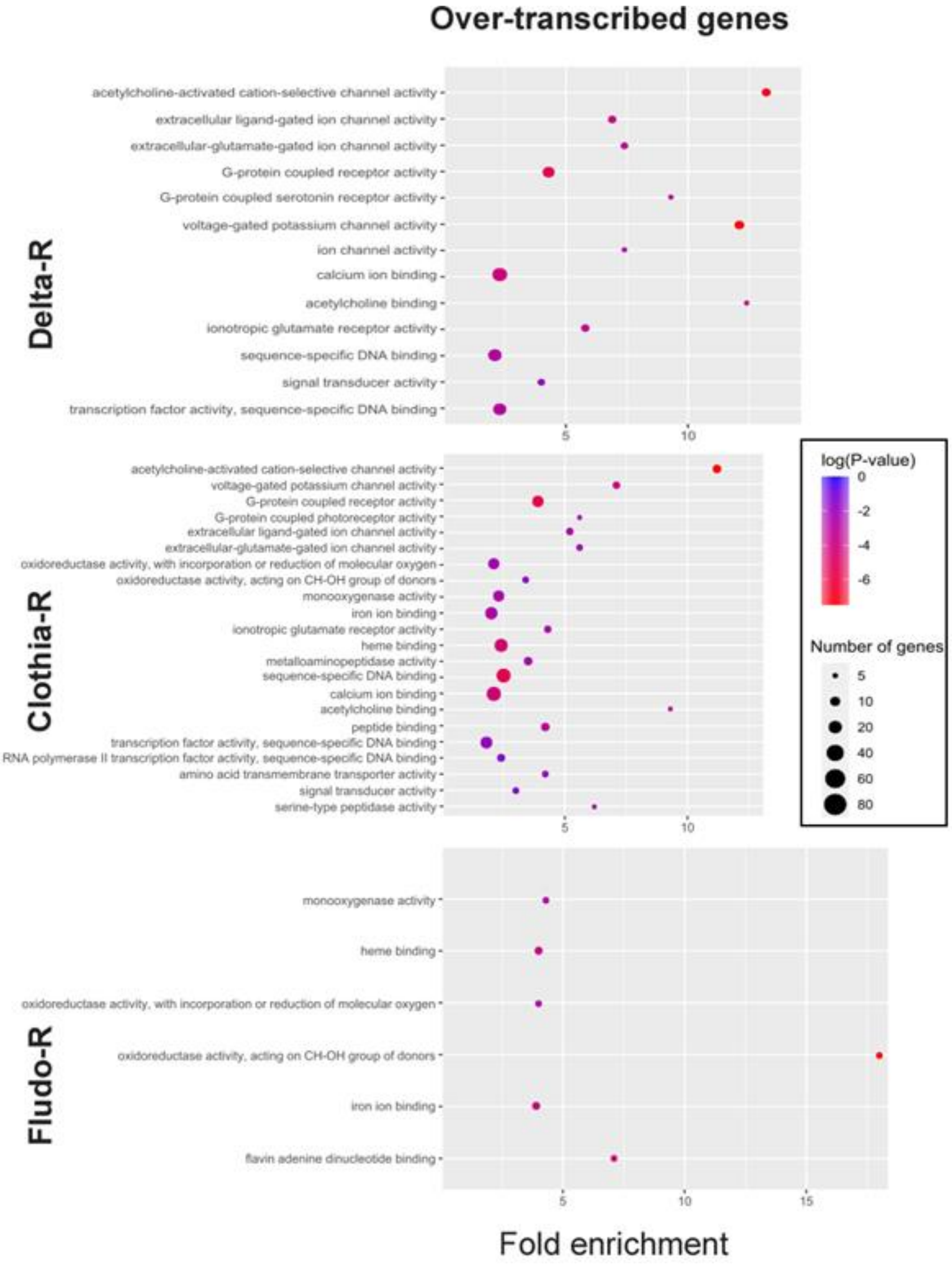

Supplementary Fig. S5 (continued)

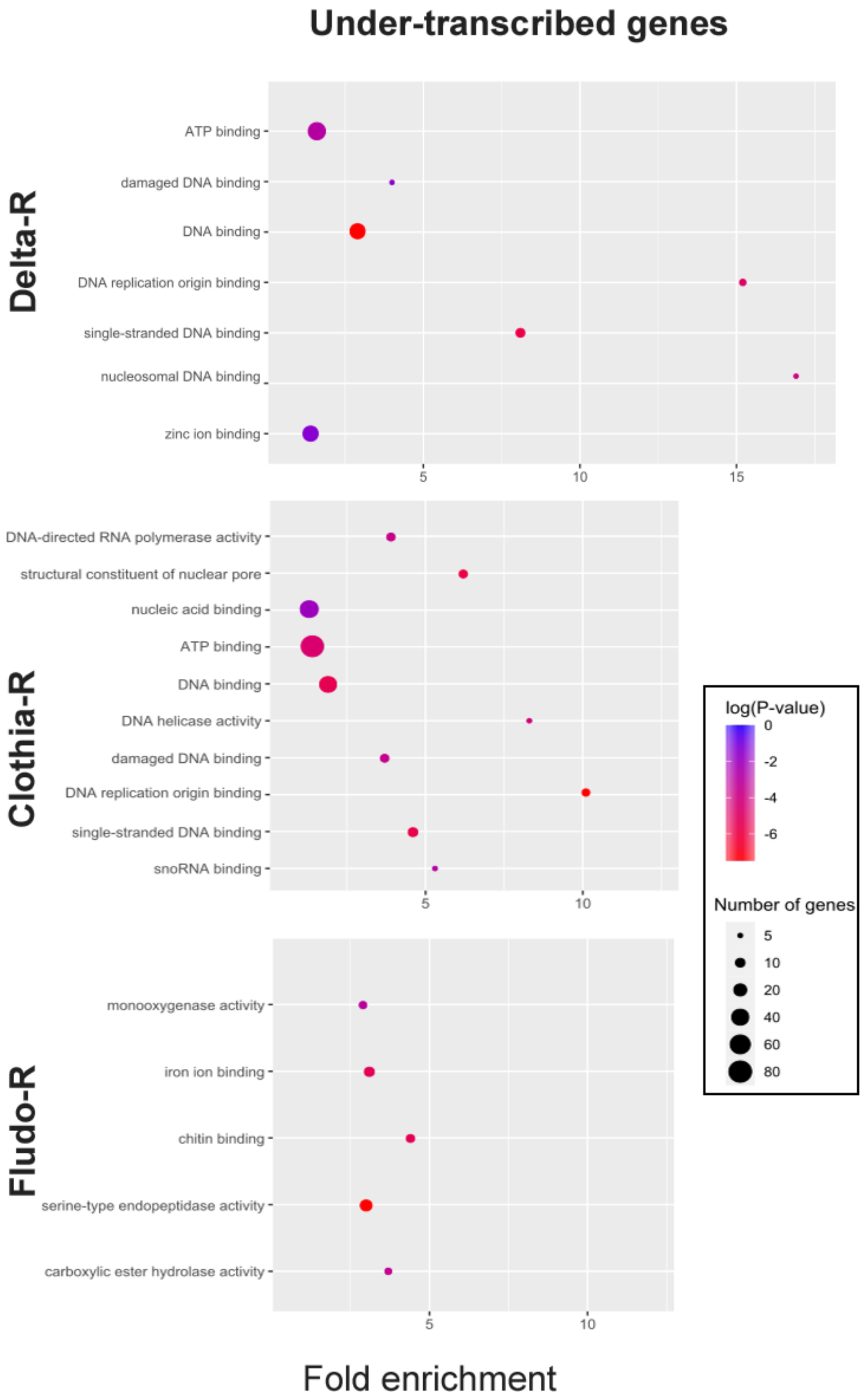

Supplementary Table S6. SNPs data obtained from RNA-seq. Only genes containing polymorphic SNPs are shown. See separated supplementary file.

Supplementary Fig. S7. Association of *CYP6M* genes transcription levels with clothianidin resistance.

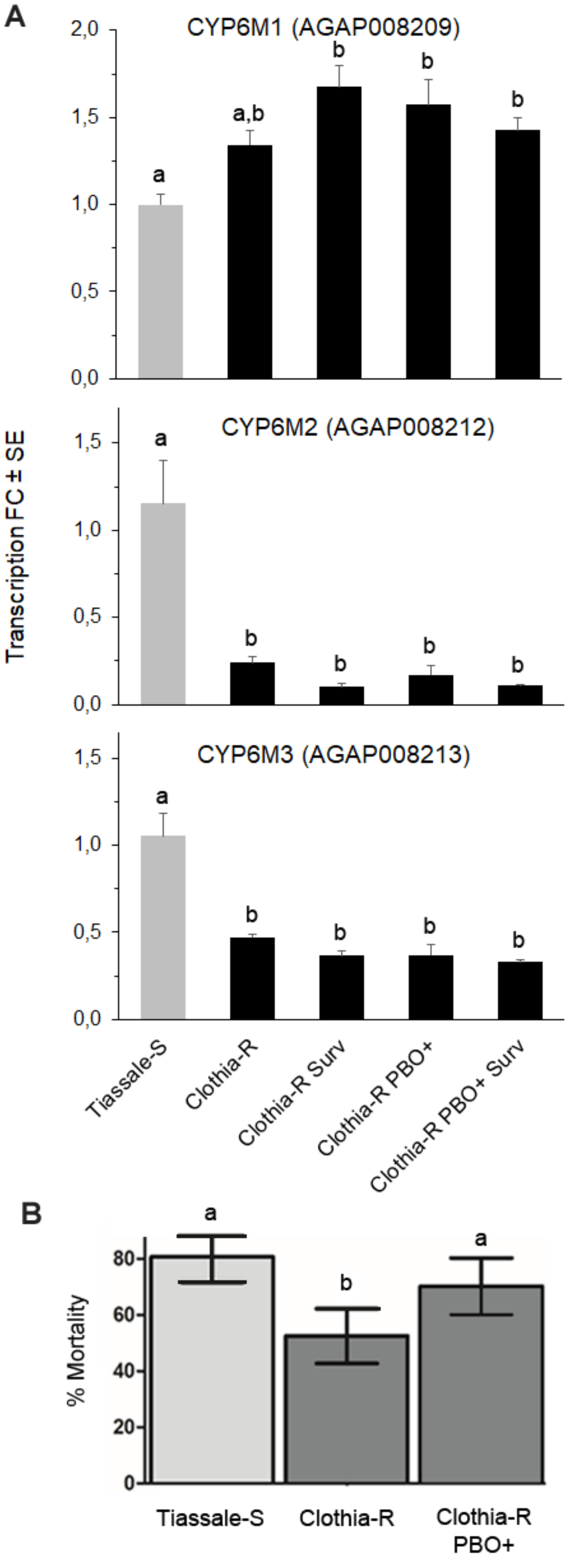

A: Transcription profiles of *CYP6M* genes in the following conditions: Tiassalé-S unexposed (Tiassalé-S); Clothia-R unexposed (Clothia-R); Clothia-R surviving to clothianidin exposure (Clothia-R Surv); Clothia-R exposed to PBO (Clothia-R PBO+); Clothia-R exposed to PBO and surviving to clothianidin exposure (Clothia-R PBO+ Surv). Mosquitoes from all conditions were sampled at the same time corresponding to 72h after clothianidin exposure (6-days old). Transcription profiles were obtained by RT-qPCR using four biological replicates per condition. Letters indicate significance between pairs of conditions ($P<0.05$).

B: Effect of PBO pre-exposure on the survival of Clothia-R individuals to clothianidin. Mosquitoes were exposed to 4% PBO for 1h prior exposure to 0.45 µg/mL clothianidin for 1h. Mortality was recorded 72h after exposure. Letters indicate significance between conditions ($P<0.05$).

Supplementary Table S8. *An. gambiae* genes showing high protein homology with genes conferring resistance to neonicotinoids in other insect species.

Species	Gene name	References	Degree of evidence	*An. gambiae* orthologs (BlastP score)
Drosophila melanogaster	*CYP6G1*	Joußen et al 2008; Sparks et al 2012; Le Goff et al 2003	Confers resistance to neonicotinoids in transgenic flies, metabolizes imidacloprid	CYP6P1 (320), CYP6P4 (318), CYP6P3 (315), CYP6M3 (312), CYP6Y2 (300)
	CYP12D1	Le Goff et al 2003	Confers resistance to neonicotinoids in transgenic flies	CYP12F1 (372)
	CYP6A8	Le Goff et al 2003	Confers resistance to neonicotinoids in transgenic flies	CYP6M4 (460), CYP6M3 (459), CYP6Y1 (456), CYP6P4 (446), CYP6Y2 (428), CYP6M1 (425), CYP6P3 (416), CYP6P1 (390) CYP6AA2 (373), CYP6Z2 (302), CYP6Z3 (300)
Bemisia tabaci	*CYP6CM1*	Karunker et al 2009	Confers resistance to neonicotinoids, metabolizes imidacloprid	CYP6M3 (309)
Myzus persicae	*CYP6CY3*	Bass et al 2013	Confers resistance to neonictoninoids in transgenic flies, metabolizes imidacloprid in vitro	CYP6M4 (376), CYP6Y1 (373), CYP6M3 (354), CYP6Y2 (353), CYP6P3 (351), CYP6P4 (350), CYP6M1 (342), CYP6AA2 (313), CYP6P1 (304)
Nilaparvata lugens	*CYP6ER1*	Pang et al 2016, Bao et al 2016, Hamada et al 2020,	Confers resistance to neonicotinoids in transgenic flies, metabolizes imidacloprid in vitro	CYP6M3 (313), CYP6P4 (301)
	CYP6AY1	Bao et al 2016	Confers resistance to neonicotinoids, metabolizes imidacloprid in vitro	CYP6M3 (385), CYP6M4 (385)
Aedes aegypti	*CYP6BB2*	Riaz et al 2012	Associated with resistance to neonicotinoids and imidacloprid metabolism	CYP6P4 (576), CYP6P3 (554), CYP6P1 (498), CYP6M3 (469), CYP6M4 (450), CYP6Y2 (414), CYP6Y1 (406), CYP6M1 (390), CYP6AA2 (372), CYP6Z2 (322), CYP6Z3 (310), CYP6Z1 (304)
	CYP6N12	Riaz et al 2012	Associated with resistance to neonicotinoids and imidacloprid metabolism	CYP6M3 (588), CYP6M4 (561), CYP6Y1 (523), CYP6Y2 (520), CYP6M1 (516), CYP6P4 (510), CYP6P3 (489), CYP6P1 (452), CYP6AA2 (416), CYP6Z2 (352), CYP6Z3 (347), CYP6Z1 (339)

Supplementary Table S9. Primers used for RT-qPCR.

Target gene	Forward primer (5' to 3')	Reverse primer (5' to 3')	Product lenght (bp)
RPS7 (AGAP010592)*	TTCAACAACAAGAAGGCGATCA	ACGGGTCTGTACCTTCTGGA	149
CYP6M1 (AGAP008209)	TTTTGAAAGAATCCCTACGCAAG	GCTCGGGAAATAGACTCGCA	162
CYP6M2 (AGAP008212)	GCACAACGGAGAGATGTCGT	TGCGCAGTGATTCATTAAGAATTTG	77
CYP6M3 (AGAP008213)	GAGACATCTTCCACGCTGCT	TGCGAAGACTCTCCTTCAGT	178

* housekeeping gene used for normalization

Supplementary Fig. S9. Amino acid sequence of the two CYP6M1 variants identified in the Tiassalé-S and Clothia-R lines and used for protein modelling. For each variant, the amino acids in grey boxes differed by more than 50% frequency as compared to the reference genome AgamP4.12 sequence (AGAP008209). Amino acids in red are those supported by an allele frequency variation >50% between the Tiassalé-S line and the Clothia-R line.

An. gambiae CYP6M1 (Tiassalé-S line)

```
MWFPTIEVLVALLALLGGAVYFIVRKQSYWKERGVPHPKPTFFFGSFKDAGTKIHFTEEVERHYAIYKGKHPFIGVYMLTTPV
VLPLDLELIKAIFVKDFQYFHDRGTYYNEKHDPLTAHLFNLEGQKWRNLRNKMTPTFTSGKMKMMFPTVVAAGQQLRDFMEEN
VQKHGEMELKDVMARYTTDVIGTCAFGIECNSMRDPDAEFRAMGKLFMERQPSQFVNMMVQFSPKLSRLLGIRFIDKEVSAFF
LKVVRDTIDYRVKNGIQRNDFMDLMIRMLQNTENPEEALTFNEVAAQAFVFFFAGFETSSTLLTWTLYELALNPEVQEKGRQC
VQEVLAKHNGEMTYDAIHDMKYLDQILKESLRKYPPVPLHFRMTAQNYRVPDTDSVIEAGTMLFIPIFSIQRDASLFPEPEKF
DPERFSAEEEAKRHPFAWTPFGEGPRVCIGLRFGMMQARIGLAYLLQGFSFAPYEKTSIPMKFITNSFILGPREGLWLKVNKL
ESKQG
```

An. gambiae CYP6M1 (Clothia-R line)

```
MWFPTIEVLVALLALLGGAVYFIVRKQSYWKERGIPHPKPTFFFGSFKDAGTKIHFTEEVERHYALYKGKHPFIGVYLLTTPV
VLPLDLELIKAIFVKDFQYFHDRGTYYNEKHDPLTAHLFNLEGQKWRNLRNKMTPTFTSGKMKMMFPTVVAAGQQLRDFMEEN
VQKHDEMELKDVMARYTTDVIGTCAFGIECNSMRDPDAEFRAMGKLFMDRQPSQFVNMMVQFSPKLSRLLGIRLIDKEVSAFF
LKVVRDTIDYRVKNGIQRNDFMDLMIRMLQNTENPEEALTFNEVAAQAFVFFFAGFETSSTLLTWTLYELALNPEVQEKGRQC
VQEVLAKHNGEMTYEAIHEMKYLDQILKESLRKYPPVPLHFRTTSQNYRVPDTDSVIEAGTMLLIPIFAIQRDASLFPEPEKF
DPERFSAEEEAKRHPFAWTPFGEGPRVCIGLRFGMMQARIGLAYLLQGFSFAPYEKTSIPMKFITNSFILGPREGLWLKVNKL
ESKQG
```
